# Development and Validation of a Prediction Model for Need for Massive Transfusion During Surgery Using Intraoperative Hemodynamic Monitoring Data

**DOI:** 10.1001/jamanetworkopen.2022.46637

**Published:** 2022-12-14

**Authors:** Seung Mi Lee, Garam Lee, Tae Kyong Kim, Trang Le, Jie Hao, Young Mi Jung, Chan-Wook Park, Joong Shin Park, Jong Kwan Jun, Hyung-Chul Lee, Dokyoon Kim

**Affiliations:** 1 Department of Obstetrics and Gynecology, Seoul National University College of Medicine, Seoul, Korea; 2 Department of Obstetrics and Gynecology, Seoul National University Hospital, Seoul, Korea; 3 Department of Biostatistics, Epidemiology and Informatics, Perelman School of Medicine, University of Pennsylvania, Philadelphia; 4 Department of Anesthesiology and Pain Medicine, Seoul Metropolitan Government Seoul National University Boramae Medical Center, Seoul, Korea; 5 Department of Anesthesiology and Pain Medicine, Seoul National University College of Medicine, Seoul, Korea; 6 Institute for Biomedical Informatics, University of Pennsylvania, Philadelphia

## Abstract

**Importance:**

Massive transfusion is essential to prevent complications during uncontrolled intraoperative hemorrhage. As massive transfusion requires time for blood product preparation and additional medical personnel for a team-based approach, early prediction of massive transfusion is crucial for appropriate management.

**Objective:**

To evaluate a real-time prediction model for massive transfusion during surgery based on the incorporation of preoperative data and intraoperative hemodynamic monitoring data.

**Design, Setting, and Participants:**

This prognostic study used data sets from patients who underwent surgery with invasive blood pressure monitoring at Seoul National University Hospital (SNUH) from 2016 to 2019 and Boramae Medical Center (BMC) from 2020 to 2021. SNUH represented the development and internal validation data sets (n = 17 986 patients), and BMC represented the external validation data sets (n = 494 patients). Data were analyzed from November 2020 to December 2021.

**Exposures:**

A deep learning–based real-time prediction model for massive transfusion.

**Main Outcomes and Measures:**

Massive transfusion was defined as a transfusion of 3 or more units of red blood cells over an hour. A preoperative prediction model for massive transfusion was developed using preoperative variables. Subsequently, a real-time prediction model using preoperative and intraoperative parameters was constructed to predict massive transfusion 10 minutes in advance. A prediction model, the massive transfusion index, calculated the risk of massive transfusion in real time.

**Results:**

Among 17 986 patients at SNUH (mean [SD] age, 58.65 [14.81] years; 9036 [50.2%] female), 416 patients (2.3%) underwent massive transfusion during the operation (mean [SD] duration of operation, 170.99 [105.03] minutes). The real-time prediction model constructed with the use of preoperative and intraoperative parameters significantly outperformed the preoperative prediction model (area under the receiver characteristic curve [AUROC], 0.972; 95% CI, 0.968-0.976 vs AUROC, 0.824; 95% CI, 0.813-0.834 in the SNUH internal validation data set; *P* < .001). Patients with the highest massive transfusion index (ie, >90th percentile) had a 47.5-fold increased risk for a massive transfusion compared with those with a lower massive transfusion index (ie, <80th percentile). The real-time prediction model also showed excellent performance in the external validation data set (AUROC of 0.943 [95% CI, 0.919-0.961] in BMC).

**Conclusions and Relevance:**

The findings of this prognostic study suggest that the real-time prediction model for massive transfusion showed high accuracy of prediction performance, enabling early intervention for high-risk patients. It suggests strong confidence in artificial intelligence-assisted clinical decision support systems in the operating field.

## Key Points

**Question:**

Can intraoperative hemodynamic monitoring data be used in real time to develop a prediction model for the need for massive transfusion during surgery?

**Findings:**

In this prognostic study of 17 986 patients, the real-time prediction model for massive transfusion using preoperative and intraoperative parameters was associated with significantly improved performance (area under the receiver operating characteristic curve = 0.972) over the benchmark model (area under the curve = 0.824) that used only preoperative variables. Patients with the highest massive transfusion index (ie, >90th percentile) had a 47.5-fold increased risk for massive transfusion compared with those with a lower massive transfusion index (ie, <80th percentile).

**Meaning:**

These findings suggest that early identification of the need for massive transfusion may allow timely intervention for high-risk patients during surgery.

## Teaser

This prognostic study assesses a real-time prediction model for massive transfusion during surgery based on the incorporation of preoperative data and intraoperative hemodynamic monitoring data.

## Introduction

Massive hemorrhage is the leading cause of death in a variety of clinical settings, such as trauma in military, civilian settings, or high-risk surgery.^1,2,3^ Management for severe hemorrhage consists of both acute bleeding control and supplementation for intravascular volume and blood component deficiencies, usually with blood product transfusion. Therefore, both hemorrhage control and massive transfusion are important components in the management of uncontrolled massive hemorrhage. Because massive transfusion involves the preparation of a large amount of blood products and additional medical personnel for a team-based approach, early prediction of massive transfusion during surgery can be helpful for appropriate management of massive hemorrhage. Indeed, delayed initiation of appropriate transfusion has been reported to be associated with increased mortality and/or morbidities.^4^

Despite the clinical significance of acute hemorrhage due to its life-threatening characteristics and the need for urgent preparation of massive transfusion to enable optimal management, the development of a prediction model for massive transfusion during surgery is lacking. Several studies have tried to predict massive transfusion during specific types of high-risk procedures, such as liver transplantation,^5,6,7^ cardiac surgery,^8,9,10^ placenta previa,^11,12^ and spine surgery,^13^ but the performances of the prediction models were low, with an area under the receiver operating characteristic curve (AUROC) of 0.65 to 0.84. These low predictive powers may originate from the fact that models developed in previous studies were solely based on preoperative factors without incorporating dynamic intraoperative parameters.

In the literature, information regarding the usefulness of intraoperative features in the prediction of massive transfusion is lacking. The absence of large data sets from intraoperative records, including waveforms, has hampered research using perioperative features.^14^ Although several detailed data sets from electronic health record systems have been available over the past decade, most of these data sets are based on monitoring systems in intensive care units.^15,16,17,18,19,20^ To date, a large data set, including intraoperative data, remains unavailable. Since 2016, Seoul National University Hospital (SNUH) has collected vital sign data from surgical patients with Vital Recorder, a free software that collects high-resolution time-synchronized data of vital signs from various medical devices.^21^ In recent years, we have published several reports using this database, named VitalDB, with hundreds or thousands of patients.^22,23^ Currently, data from more than 50 000 patients have been collected, enabling research on rare complications, such as those requiring massive transfusion.

In cases with acute hemorrhage requiring transfusion, patients’ volume status can result in a change in hemodynamic parameters. Subtle changes in patients’ vital signs may be assumed to occur before a definite change that physicians can recognize. Machine learning is a type of artificial intelligence that can learn nonlinear relationships between variables through pattern recognition from many data sets.^24,25,26^ Therefore, an appropriate algorithm developed by machine learning may be able to detect early alterations in patterns of patients’ continuous hemodynamic signals prior to an event necessitating massive transfusion, which may sometimes be too large to be processed by humans.

In the current study, we developed a model for real-time prediction of massive transfusion using intraoperative hemodynamic monitoring data by applying deep learning methods. Our hypothesis was that the machine learning model for the prediction of massive transfusion during surgery would show good predictive power in internal and external validation.

## Methods

The registry of intraoperative vital signs data (VitalDB) used in this study was approved by the institutional review board (IRB) of Seoul National University Hospital and registered at the public clinical trial registration site (ClinicalTrials.gov, NCT02914444). The IRBs of SNUH and BMC also approved the retrospective analysis of the registry for this study. The study was conducted in accordance with the principles of the Declaration of Helsinki. The IRB waived the requirement for informed consent because of the retrospective nature of the study. We followed the Transparent Reporting of a Multivariable Prediction Model for Individual Prognosis or Diagnosis (TRIPOD) reporting guideline.^27^

### Data Source

**Figure 1.**
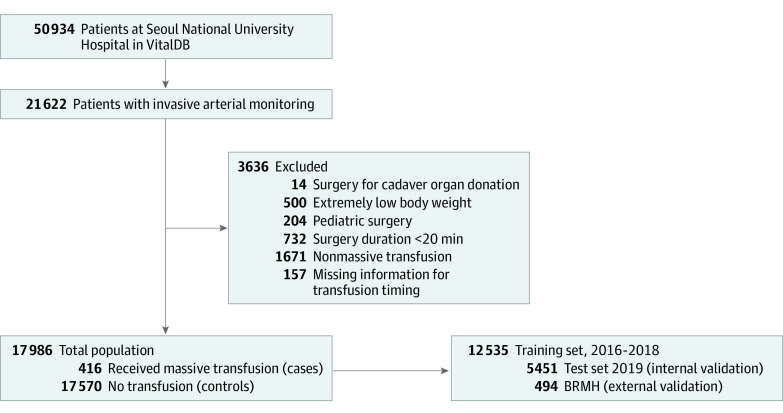
Flow Diagram of Study Population

In this prognostic study, the study population consisted of patients whose physiological data were registered at the VitalDB between August 2016 and December 2019 at SNUH (Figure 1), which is tertiary care in the Republic of Korea. Among these patients at SNUH (n = 50 934), the current study population included those with available intra-arterial blood pressure monitoring records (n = 21 622). Patients undergoing surgical procedures for deceased organ donation, surgical procedures less than 20 minutes, or pediatric surgical procedures (<18 years old), and patients with extremely low body weights (<30 kg) were excluded from the study population. Prior to analysis, cases at SNUH were divided into development and internal validation data sets according to the year of surgery. The development data set included 12 535 patients who underwent surgery between 2016 and 2018, and the internal validation data set included 5451 patients who underwent surgery in 2019 (Figure 1).

For further external validation, we evaluated the prediction model on a geotemporally external validation set consisting of 494 patients who underwent surgery between 2020 and 2021 in Boramae Medical Center (BMC), which is secondary care in Republic of Korea. BMC has started to collect data sets from intraoperative records using the same protocol as SNUH since 2020.

### Outcome Definition

Massive transfusion was defined as transfusion of 3 or more units of red blood cells over an hour.^28,29,30^ Documentation of the start time of each transfused blood unit is a routine practice, which can be retrieved from electronic health records (EHRs). Patients who received intraoperative transfusion but not massive transfusion or those without documentation of the transfusion start time were excluded from the analysis.

### Preoperative Features

Preoperative features, such as patient demographic information and preoperative laboratory results were also collected from EHRs. The preoperative features used for model construction were (1) baseline demographic factors, including age, sex, weight, height, underlying medical diseases such as hypertension, diabetes, tuberculosis, liver disease, chronic obstructive pulmonary disease, asthma, heart disease, thyroid disease, renal disease, hematologic disease, vascular disease, neurologic disease or pregnancy, and the American Society of Anesthesiologists physical status classification; (2) features associated with the operation, including department of surgery and the planned type of anesthesia; and (3) preoperative laboratory tests, including hemoglobin, hematocrit, platelet counts, albumin, alanine aminotransferase, aspartate aminotransferase, electrolytes, glucose, high-density lipoprotein, blood urea nitrogen, creatinine, prothrombin time, activated partial thromboplastin time, and the estimated glomerular filtration rate.

### Intraoperative Features

Among intraoperative vital sign records from VitalDB, 3 types of data sources were utilized: (1) intra-arterial blood pressure waveforms; (2) oxygen saturation (Spo_2_) from the patient monitor (Solar 8000 M, GE Healthcare); (3) The ST segment elevation read from electrocardiography waveforms.

**Figure 2.**
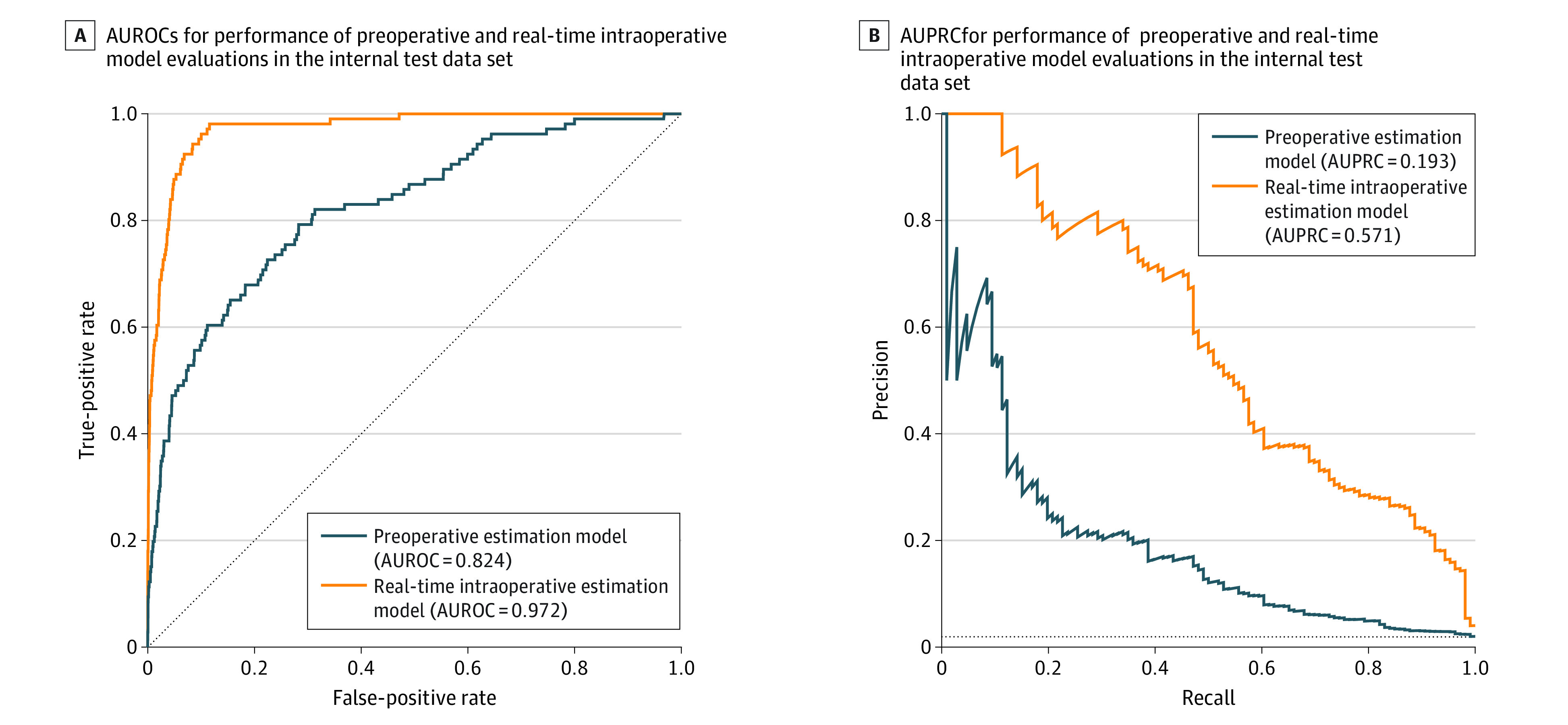
The Performance of Preoperative and Real-Time Intraoperative Prediction Model Evaluations in the Internal Test Data Set A, Area under the receiver characteristic curve (AUROC). B, Area under the precision-recall curve (AUPRC).

From the intra-arterial waveform, we used the waveform factorization algorithm publicly provided by VitalDB. The following measures were calculated from the arterial pressure waveform (Figure 2): (1) blood pressure (BP): systolic BP, diastolic BP, and the mean BP; (2) heart rate; and (3) the area under the arterial waveform in each cardiac beat.

During the operation, hematocrit levels were measured at the discretion of the anesthesiologist. The baseline hematocrit levels and intraoperative hematocrit levels were also used to develop the prediction model.

### Designation of the Observation Window

We set up the observation window and prediction period to conceptualize the temporal input length and expected time point of prediction (Figure 2). For the development of the prediction model, the risk for massive transfusion was calculated using intraoperative features, which were extracted from intraoperative vital sign records during a 10-minute observation window. At the end of the observation window, the prediction model calculated the risk of massive transfusion after the prespecified prediction period.

For cases of massive transfusion, the model for predicting massive transfusion 10 minutes in advance used intraoperative features extracted from intraoperative vital sign records between 20 minutes and 10 minutes before the start of massive transfusion (ie, prediction period, Δ*t* = 10 minutes). For controls without massive transfusion, the endpoint of the observation window was randomly selected, and an intraoperative vital sign record data set between 10 minutes and 0 minutes before the endpoint was used for extraction of intraoperative parameters (observation window, 10 minutes) (Figure 2).

To explore the optimal prediction timing, we also set the prediction period to 15 minutes or 20 minutes prior to the onset of massive transfusion (prediction period, Δ*t* = 15 or 20 minutes). Therefore, the model for predicting massive transfusion 15 minutes in advance was developed using intraoperative vital sign records between 25 minutes and 15 minutes before the start of massive transfusion (ie, prediction period, Δ*t* = 15 minutes; observation window, 10 minutes), and the model for predicting massive transfusion 20 minutes in advance used intraoperative vital sign records between 30 minutes and 20 minutes before the start of massive transfusion (ie, prediction period, Δ*t* = 20 minutes; observation window, 10 minutes).

### Development of the Model and Statistical Analysis

Cases with missing clinical information or invalid arterial waveforms were excluded from the analysis. For the development of a preoperative prediction model, we used logistic regression, *l*_1_ and *l*_2_-regularized logistic regression (Lasso^31^ and Ridge,^32^ respectively), random forest, and gradient boosting. Logistic regression is commonly used in classification tasks due to its simplicity. It transforms the linear combination of input predictors into a probability that can be mapped to a binary class. In our study, binary class refers to patients with or without massive transfusion events. Random forest and gradient boosting are tree-based algorithms in which multiple base classifiers are aggregated to produce final predictions.^33,34^

For the development of a real-time intraoperative prediction model using intraoperative parameters, we used a gated recurrent unit (GRU).^35^ The GRU has a feedback loop that takes 1 element at a time to update its hidden state and return output. The architectural uniqueness enables analysis of sequential intraoperative features and produces regularized representations. Finally, the representation was integrated with preoperative variables to construct a prediction model.

The developed real-time intraoperative prediction model calculated the risk for massive transfusion, which was designated the massive transfusion index. The performance of the massive transfusion index developed from the real-time intraoperative prediction model was compared with that of the preoperative prediction model, using the AUROC and the area under the precision-recall curve (AUPRC). For binary classification of patients according to the calculated risk from these machine learning algorithms, the optimal cutoff value was estimated using the Youden Index, which maximizes the difference between the true-positive rate and false-positive rate over all possible cutoff values.^36^ Multiple evaluation metrics, such as sensitivity, specificity, positive predictive value (PPV), and negative predictive value (NPV), were measured to show performance discrepancies due to the imbalanced sample ratio.

For model assessment, we divided the patients from SNUH into development and internal validation data sets according to the year of the operation (ie, surgery between 2016 and 2018), as previously described. The development data set (ie, surgery in 2019) was used to construct prediction models using stratified 5-fold cross-validation with a repetition of 10 times. We selected the best model showing the highest AUROC in the internal validation data set and evaluated the prediction performance with external validation data sets (ie, surgery between 2020 and 2021). Statistical prediction improvement was evaluated with the DeLong test, which provides a 95% CI and SD of the difference between 2 AUROCs. Data were analyzed from November 2020 to December 2021.

## Results

Among 17 986 patients at SNUH (mean [SD] age, 58.65 [14.81] years; 9036 [50.2%] female) and 494 patients at BMC, 416 (2.3%) in SNUH and 11 (2.2%) in BMC underwent massive transfusion during the operation (mean [SD] duration of operation, 171.0 [105.0] minutes in SNUH and 149.3 [94.1] minutes in BMC). Among 50 934 patients who underwent surgery during the study period at SNUH, intra-arterial blood pressure waveform data were monitored and were available for 21 622 patients (Figure 1). Among these, we excluded patients undergoing surgical procedures for deceased organ donation (14 patients), surgical procedures less than 20 minutes (732 patients), and pediatric surgical procedures (204 patients), or those with extremely low body weight (500 patients) from the study population. After excluding patients with nonmassive transfusion (1671 patients), those without transfusion time documentation (157 patients), or those with invalid arterial waveforms (358 patients), the remaining 17 986 patients were analyzed in the final analysis. Then, the SNUH study population was divided into development and internal validation data sets (12 535 patients [69.7%] and 5451 patients [30.3%]). Further external validation was performed in the BMC external validation data set (494 patients). Table 1 shows the baseline clinical features and primary outcomes of the study population. The incidence rates of massive transfusion during surgery in the SNUH development data set, SNUH internal validation data set, and BMC external validation data set were 310 of 12 535 patients (2.5%), 106 of 5451 patients (1.9%), and 11 of 494 patients (2.2%), respectively.

**Table 1.** Baseline Clinical Features and Primary Outcomes of the Study Population

Characteristics	Cases, No. (%)		
	SNUH		BMC external validation data set (n = 494)
	Development data set (n = 12 535)	Internal validation data set (n = 5451)	
Preoperative characteristics			
Age, mean (SD), y	58.60 (14.80)	58.77 (14.83)	62.38 (14.33)
Sex			
Male	6374 (50.8)	2576 (47.3)	243 (49.2)
Female	6161 (49.1)	2875 (52.7)	251 (50.8)
BMI, mean (SD)	24.04 (3.66)	24.30 (3.77)	24.35 (4.20)
Preexisting comorbidities			
Hypertension	1878 (15.0)	747 (13.7)	167 (33.8)
Diabetes	931 (7.4)	398 (7.3)	92 (18.6)
Heart disease	616 (4.9)	257 (4.7)	57 (11.5)
Surgery information			
Surgery departments			
General surgery	5047 (40.3)	1530 (28.1)	92 (18.6)
Neurosurgery	1570 (12.5)	841 (15.4)	42 (8.5)
Thoracic surgery	2684 (21.4)	1109 (20.3)	48 (9.7)
Obstetrics and gynecology	1391 (11.1)	838 (15.4)	84 (17.0)
Orthopedics	796 (6.4)	434 (8.0)	130 (26.3)
Urologic surgery	865 (6.9)	563 (10.3)	33 (6.7)
Surgery duration, mean (SD), min	170.99 (105.03)	167.49 (104.96)	149.28 (94.10)
Anesthesia type (general)	11 920 (95.1)	5199 (95.4)	445 (90.1)
Emergency operation	608 (4.9)	279 (5.1)	30 (6.1)
Massive transfusion during surgery	310 (2.5)	106 (1.9)	11 (2.2)

Abbreviations: BMC, Boramae Medical Center; BMI, body mass index; SNUH, Seoul National University Hospital.

We first established the preoperative prediction model using preoperative features, including demographic information and preoperative laboratory results. Among various machine learning methods, the best prediction model was from the gradient boosting algorithm, with an AUROC of 0.824 in the SNUH internal validation data set (AUROC, 0.781 by logistic regression, 0.805 by Lasso, 0.781 by Ridge, and 0.787 by random forest) (Table 2).

**Table 2.** Prediction Model Evaluation in the SNUH Study Population

Prediction model	Cross-validation at SNUH development dataset (n = 12 535)		SNUH internal validation dataset (n = 5451)	
	AUROC (SD)	AUPRC (SD)	AUROC	AUPRC
**Preoperative prediction model (with preoperative variables)**				
Baseline (LR)	0.828 (0.030)	0.281 (0.04)	0.781	0.157
Baseline (Lasso)	0.837 (0.029)	0.288 (0.04)	0.805	0.169
Baseline (Ridge)	0.828 (0.030)	0.288 (0.04)	0.781	0.157
Baseline (GB)	0.839 (0.028)	0.289 (0.05)	0.824	0.193
Baseline (RF)	0.816 (0.032)	0.337 (0.05)	0.787	0.184
**Real-time intraoperative model with single intraoperative variable**				
AUAW	0.865 (0.017)	0.154 (0.010)	0.890	0.196
MBP	0.825 (0.040)	0.125 (0.028)	0.816	0.114
SBP	0.822 (0.023)	0.128 (0.016)	0.815	0.135
DBP	0.814 (0.026)	0.131 (0.019)	0.834	0.096
HCT	0.943(0.021)	0.270 (0.056)	0.957	0.521
HR	0.662 (0.248)	0.112 (0.065)	0.826	0.110
Preoperative variables and HCT	0.962 (0.005)	0.603 (0.060)	0.967	0.550
**Real-time intraoperative model with 2 intraoperative variables**				
AUAW, HR	0.873 (0.014)	0.167 (0.024)	0.887	0.189
MBP, HR	0.831 (0.029)	0.129 (0.012)	0.853	0.144
SBP, HR	0.832 (0.024)	0.130 (0.017)	0.814	0.136
DBP, HR	0.806 (0.009)	0.116 (0.007)	0.824	0.097
AUAW, HCT	0.968 (0.013)	0.354 (0.061)	0.965	0.554
MBP, HCT	0.956 (0.018)	0.295 (0.090)	0.962	0.547
SBP, HCT	0.960 (0.011)	0.277 (0.015)	0.961	0.501
DBP, HCT	0.949 (0.021)	0.285 (0.061)	0.958	0.512
SS	0.747 (0.039)	0.096 (0.014)	0.771	0.067
Preoperative variables and AUAW, HCT	0.973 (0.006)	0.629 (0.059)	0.965	0.565
**Real-time intraoperative model with addition of Spo_2_ and ST**				
MBP, HCT, Spo_2_, ST	0.959 (0.011)	0.294 (0.063)	0.962	0.537
SBP, HCT, Spo_2_, ST	0.953 (0.018)	0.300 (0.065)	0.960	0.419
DBP, HCT, Spo_2_, ST	0.955 (0.014)	0.303 (0.068)	0.960	0.504
Integral, HCT, Spo_2_, ST	0.950 (0.025)	0.289 (0.054)	0.963	0.472
Preoperative variables and AUAW, HCT, Spo_2_, ST	0.969 (0.012)	0.610 (0.059)	0.972	0.571
** Real-time intraoperative model with all available variables**				
All variables	0.955 (0.007)	0.266 (0.006)	0.954	0.401

Abbreviations: AUAW, area under the arterial waveform in each cardiac beat; AUPRC, area under the precision-recall curve; AUROC, area under the ROC curve; DBP, diastolic blood pressure; HCT, hematocrit; HR, heart rate; GB, gradient boosting; MBP, mean blood pressure; SBP, systolic blood pressure; SNUH, Seoul National University Hospital; Spo_2_, peripheral blood oxygen saturation from pulse oximeter; ST, ST segment elevation; RF, random forest.

We then evaluated whether the real-time intraoperative prediction model can improve the prediction of massive transfusion by incorporating preoperative variables with intraoperative features (Figure 2). To explore the best combination of intraoperative features, we first compared the performance of real-time intraoperative prediction models from each intraoperative feature (Table 2). Among the combinations of features, the model using area under the arterial waveform in each cardiac beat, Spo_2_, ST, and intra-operative hematocrit values combined with preoperative features showed the best performance in the internal validation data set and was designated the final prediction model (AUROC, 0.972; 95% CI, 0.968-0.976 in the SNUH internal validation data set).

The real-time intraoperative prediction model significantly outperformed the preoperative prediction model in the SNUH internal validation data set (AUROC: 0.972; 95% CI, 0.968-0.976 vs 0.824; 95% CI, 0.813-0.834; AUPRC: 0.571; 95% CI, 0.558-0.584 vs 0.193; 95% CI, 0.182-0.203; *P* < .001) (Table 2 and eFigure 1 in the Supplement). The real-time prediction model achieved a better AUPRC in the SNUH internal validation data set. The real-time prediction model also showed similar performance in the BMC external validation data set, with an AUROC of 0.943 (95% CI, 0.919-0.961) and AUPRC of 0.370 (95% CI, 0.327-0.413) (eFigure 2 in the Supplement).

When analyzing the performance with regard to prediction timing, we found that prediction of massive transfusion 15 or 20 minutes in advance showed similar performance compared with the prediction of massive transfusion 10 minutes in advance (SNUH internal validation data set: AUROC, 0.972 vs 0.969 vs 0.962 and AUPRC, 0.571 vs 0.555 vs 0.492; *P* = .60; BMC external validation data set: AUROC, 0.943 vs 0.957 vs 0.912 and AUPRC, 0.370 vs 0.458 vs 0.393; *P* = .81) (eTable 2 in the Supplement).

The final real-time intraoperative prediction model calculated the risk of massive transfusion after 10 minutes, which was named the massive transfusion index. In the SNUH study population, the risk of massive transfusion increased abruptly in the last 90th percentile of massive transfusion index (eFigure 3 in the Supplement). The model provided 47.5-fold enrichment between up to 8th and highest deciles. In the BMC study population, the model provided 9-fold enrichment between up to the 8th decile and the highest deciles (eFigure 4 in the Supplement). Most controls had a massive transfusion index less than 0.1, while the massive transfusion index of most cases was higher than 0.1 (eFigure 3 and eFigure 4 in the Supplement).

**Figure 3.**
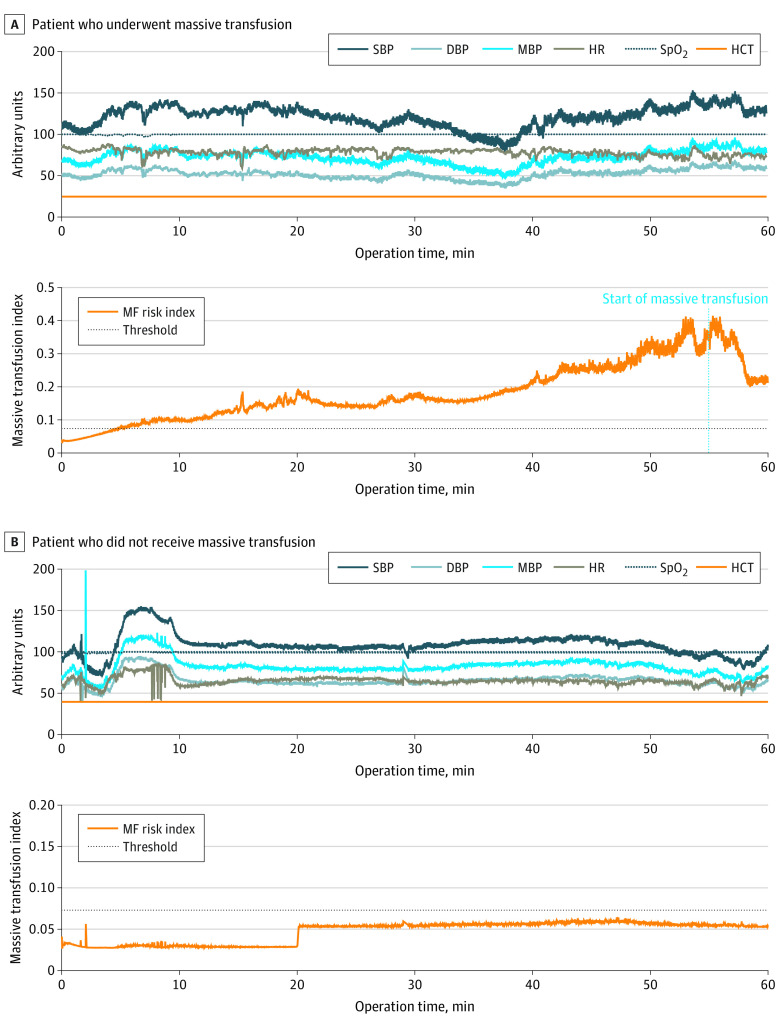
Massive Transfusion Index A, An example of a patient who underwent massive transfusion. B, An example of a patient who did not undergo massive transfusion during surgery. Abbreviations: MF, massive transfusion; DBP, diastolic blood pressure; HCT, hematocrit; HR, heart rate; MBP, mean blood pressure; Spo_2_, peripheral blood oxygen saturation (Spo_2_) from pulse oximeter; SBP, systolic blood pressure.

Figure 3 depicts an example of the massive transfusion index of patients with and without massive transfusion during surgery. At the beginning of surgery, both patients’ massive transfusion indices started at approximately 0.04. However, in the case of massive transfusion, the massive transfusion index gradually increased and exceeded the massive transfusion threshold at 4.8 minutes from the beginning of the operation, remaining between 0.24 and 0.28 at 10-minute prior to massive transfusion. In contrast, the patient with no massive transfusion had a persistent, stable massive transfusion index (less than 0.1).

We also evaluated the performance of the prediction model according to the level of urgency and type of anesthesia. In the SNUH internal validation data set, 279 of 5451 patients (5.1%) underwent emergency surgery. In addition, in the BMC external validation data set, 30 of 494 patients (6.1%) underwent emergency surgery. The real-time prediction model also showed excellent performance in both elective and emergency surgery in both study populations (AUROC, 0.975 in patients with elective surgery in SNUH internal validation data set; 0.942 in patients with emergency surgery in SNUH internal validation data set; 0.944 in patients with elective surgery in BMC external validation data set; 0.919 in patients with emergency surgery in BMC external validation data set). In SNUH internal validation data set, 5199 of 5451 patients (95.4%) underwent surgery under general anesthesia. In addition, in BMC external validation data set, 445 of 494 patients (90.1%) underwent surgery under general anesthesia. The real-time prediction model also showed excellent performance in both general anesthesia and other types of anesthesia in both study populations (AUROC, 0.973 [95% CI, 0.969-0.977]) in patients with general anesthesia in SNUH internal validation data set; 0.920 (95% CI, 0.863-0.959) in patients with regional anesthesia in SNUH internal validation data set; 0.947 (95% CI, 0.923-0.966) in patients with general anesthesia in BMC external validation data set; other types of anesthesia in BMC external validation data set could not be evaluated because there was no case with massive transfusion.

## Discussion

In this prognostic study, we examined the possibility of using intraoperative parameters from patient hemodynamic data for the prediction of massive transfusion 10 minutes in advance, allowing early intervention for high-risk patients. As a result, the real-time probability of massive transfusion could be predicted accurately 10 minutes before the start of massive transfusion, showing significantly better performance than the model using preoperative variables.

In the setting of uncontrolled massive bleeding, appropriate initiation of massive transfusion is essential for improving patient outcomes.^4^ In trauma patients with massive bleeding, several studies have highlighted the need for massive transfusion prediction.^37^ Several clinical parameters, such as blood pressure, heart rate, oxygen saturation, or the mechanism of injury, have been suggested as predictors for the need for massive transfusion.^38^

Our finding is consistent with the results from these reports for trauma patients.^37,38^ In the current study, we found the model with area under the arterial waveform in each cardiac beat, which represents the status of BP and HR, intra-operative hematocrit, Spo_2_, and ST combined with preoperative features predicted the probability of massive transfusion with the best accuracy. The importance of area under the arterial waveform in each cardiac beat is partly consistent with findings from previous studies reporting that the shock index can be used for outcome prediction in trauma patients.^39,40,41^ The shock index is defined as HR divided by systolic blood pressure and has been reported to predict patients’ hemodynamic status better than either HR or systolic blood pressure alone.^42^

In the current study, we evaluated the usefulness of real-time intraoperative hemodynamic data in predicting the need for massive transfusion. To our knowledge, the current study is the first to use intraoperative parameters in a large number of patients for the prediction of massive transfusion. Since 2016, SNUH has collected hemodynamic data in operating rooms. Although interest and/or concern has emerged regarding the adoption of machine learning algorithms in clinical practice,^43,44,45,46^ the translational machine learning approach in clinical practice has been challenging for some reasons. First, data from EHRs are heterogeneous and designed for reporting, liability, or even billing requirements rather than supplying a database for machine learning.^47^ Second, data are often stored in various systems, requiring merging and harmonization before using them as input for machine learning. Third, patients’ data in EHRs often have missing values, errors, or artifacts. Fourth, large data sets from operating rooms were not available, although several large data sets from intensive care units were available. To overcome these issues, SNUH started to use vital sign records to automatically collect time-synchronized vital sign data from various patient monitoring equipment with high resolution for research purposes. As a result, the automatic recording was successful in almost 98.5% of cases, and recently, data from more than 50 000 patients have been collected, and we could develop a prediction model for intraoperative complications, such as massive transfusion.

Our result has several clinical implications. The developed model can facilitate early recognition of the need for massive transfusion and subsequent management. The effect of early intervention has been reported in previous reports^4^ and the current model is believed to improve patient outcomes. Recently, artificial intelligence (AI) has emerged as an adjuvant tool for clinical decision-making in the setting of huge information.^43,44,45,46^ In the current study, we showed that machine learning-developed algorithms successfully classified patients as high risk and in need of attention and intervention and showed the possibility of AI-CDSS (clinical decision support system) in clinical practice. In clinical circumstances, the developed model for the need for massive transfusion may allow the anesthesia team for appropriate intervention, including preparation of a large amount of blood products, call for additional medical personnel for a team-based approach, and timely initiation of appropriate transfusion. In addition, the operating team may pay attention to surgical procedures to reduce the amount of active bleeding and call for additional medical personnel for optimal surgery. However, for implementation in routine practice, further studies are needed regarding the effectiveness of the developed model in clinical settings.

Another impactful application is the use of a machine learning-based algorithm to interpret continuous data sets from clinical information. Along with the improvement in artificial intelligence, machine learning is believed to be powerful in settings where data or signals are produced faster than humans can interpret. During surgery, extensive input information arises from patients, which can sometimes be enormous for clinicians. In addition, a patient’s vital signs change continuously during a surgical procedure, and physicians must interpret vital signs in the context of serial changes. In the current study, we used a recurrent deep learning model to analyze temporal dynamic data. The adoption of a recurrent machine learning algorithm allowed predictions to change over time (real-time prediction) based on changes in patients’ vital signs during surgery, which facilitated the detection of changes in the clinical state and early decision-making.

### Limitations

This study has several limitations. First, our prediction model is developed from a retrospective database with inherent limitations, such as selection bias and the influence of the interaction of physicians and the health system. However, we used a data set containing information from consecutive patients who underwent surgery in an operating room, reducing the possibility of selection bias. Additionally, model performance was validated using data from the patients at different time points and different locations. Second, the results of the current study were based on a database from South Korea, and further studies are needed to evaluate the model’s effectiveness in other ethnicities or races. Third, in the prediction model, we were not able to consider the amount of heavy bleeding, which was difficult to quantify.

## Conclusions

In this diagnostic study, we developed a real-time intraoperative prediction model for massive transfusion with high accuracy. Our results suggest the possibility of early intervention for high-risk patients. Future prospective studies are needed to determine the clinical usefulness of the developed model, for example, the extent to which early intervention for patients with massive hemorrhage can improve clinical outcomes.

## Notes

Supplement. **eTable 1.** Prediction Model Evaluation in SNUH Study Population **eTable 2.** Performance of the Final Prediction Model Across Prediction Periods **eFigure 1.** Overview of Data Engineering and Integration **eFigure 2.** The Performance of Preoperative and Real-Time Intraoperative Prediction Model in BMC External Validation Dataset **eFigure 3.** Massive Transfusion Index Analysis in SNUH Study Population **eFigure 4.** Massive Transfusion Index Analysis in BMC Study Population
