# Supplementary material for: Development and Validation of a Prediction Model for Need for Massive Transfusion During Surgery Using Intraoperative Hemodynamic Monitoring Data
Source: JAMA Netw Open. 2022 Dec 14;5(12):e2246637. doi: 10.1001/jamanetworkopen.2022.46637 (PMC9856486; doi:10.1001/jamanetworkopen.2022.46637)
Supplement: Supplement. — eTable 1. Prediction Model Evaluation in SNUH Study Population eTable 2. Performance of the Final Prediction Model Across Prediction Periods eFigure 1. Overview of Data Engineering and Integration eFigure 2. The Performance of Preoperative and Real-Time Intraoperative Prediction Model in BMC External Validation Dataset eFigure 3. Massive Transfusion Index Analysis in SNUH Study Population eFigure 4. Massive Transfusion Index Analysis in BMC Study Population [file jamanetwopen-e2246637-s001.pdf]

Supplemental Online Content

Lee SM, Lee G, Kim TK, et al. Development and validation of a prediction model for need for massive transfusion during surgery using intraoperative hemodynamic monitoring data. *JAMA Netw Open*. 2022;5(12):e2246637.
doi:10.1001/jamanetworkopen.2022.46637

eTable 1. Prediction Model Evaluation in SNUH Study Population
eTable 2. Performance of the Final Prediction Model Across Prediction Periods
eFigure 1. Overview of Data Engineering and Integration
eFigure 2. The Performance of Preoperative and Real-Time Intraoperative Prediction Model in BMC External Validation Dataset
eFigure 3. Massive Transfusion Index Analysis in SNUH Study Population
eFigure 4. Massive Transfusion Index Analysis in BMC Study Population

This supplemental material has been provided by the authors to give readers additional information about their work.

© 2022 Lee SM et al. *JAMA Network Open.*

eTable 1. Prediction Model Evaluation in the SNUH Study Population

	Cross-validation at SNUH development dataset (n=12,535)		SNUH internal validation dataset (n=5,451)	
	AUROC (sd)	AUPRC (sd)	AUROC	AUPRC
Pre-operative prediction model (with pre-op variables)				
baseline (LR)	0.828 (0.030)	0.281 (0.04)	0.781	0.157
baseline (Lasso)	0.837 (0.029)	0.288 (0.04)	0.805	0.169
baseline (Ridge)	0.828 (0.030)	0.288 (0.04)	0.781	0.157
baseline (GB)	0.839 (0.028)	0.289 (0.05)	0.824	0.193
baseline (RF)	0.816 (0.032)	0.337 (0.05)	0.787	0.184
Real-time intra-operative model with single intra-operative variable				
au_aW	0.865 (0.017)	0.154 (0.010)	0.890	0.196
mBP	0.825 (0.040)	0.125 (0.028)	0.816	0.114
sBP	0.822 (0.023)	0.128 (0.016)	0.815	0.135
dBP	0.814 (0.026)	0.131 (0.019)	0.834	0.096
hct	0.943(0.021)	0.270 (0.056)	0.957	0.521
hr	0.662 (0.248)	0.112 (0.065)	0.826	0.110
**Pre-operative variables + hct**	**0.962 (0.005)**	**0.603 (0.060)**	**0.967**	**0.550**
Real-time intra-operative model with 2 intra-operative variables				
au_aW, hr	0.873 (0.014)	0.167 (0.024)	0.887	0.189
mBP, hr	0.831 (0.029)	0.129 (0.012)	0.853	0.144
sBP, hr	0.832 (0.024)	0.130 (0.017)	0.814	0.136
dBP, hr	0.806 (0.009)	0.116 (0.007)	0.824	0.097
au_aW, hct	0.968 (0.013)	0.354 (0.061)	0.965	0.554
mBP, hct	0.956 (0.018)	0.295 (0.090)	0.962	0.547
sBP, hct	0.960 (0.011)	0.277 (0.015)	0.961	0.501
dBP, hct	0.949 (0.021)	0.285 (0.061)	0.958	0.512
SS	0.747 (0.039)	0.096 (0.014)	0.771	0.067
**Pre-operative variables + au_aW, hct**	**0.973 (0.006)**	**0.629 (0.059)**	**0.965**	**0.565**
Real-time intra-operative model with addition of SpO_2 and ST				
mBP, hct, SpO_2, ST	0.959 (0.011)	0.294 (0.063)	0.962	0.537
sBP, hct, SpO_2, ST	0.953 (0.018)	0.300 (0.065)	0.960	0.419
dBP, hct, SpO_2, ST	0.955 (0.014)	0.303 (0.068)	0.960	0.504
integral, hct, SpO_2, ST	0.950 (0.025)	0.289 (0.054)	0.963	0.472
**Pre-operative variables + au_aW, hct, SpO_2, ST**	**0.969 (0.012)**	**0.610 (0.059)**	**0.972**	**0.571**
Real-time intra-operative model with all available variables				
**All variables**	**0.955 (0.007)**	**0.266 (0.006)**	**0.954**	**0.401**

Abbreviations: au_aW, area under the arterial waveform in each cardiac beat; AUPRC, area under the precision-recall curve; AUROC, area under the ROC curve; MF, massive transfusion; dBP, diastolic blood pressure; hct, hematocrit; HR, heart rate; GB, gradient boosting; GRU, gated recurrent units; logit, logistic regression; mBP, mean blood pressure; sBP, systolic blood pressure; sd, standard deviation; SNUH, Seoul National University Hospital; SpO2, peripheral blood oxygen saturation from pulse oxymeter; ST, ST segment elevation; RF, random forest.

© 2022 Lee SM et al. *JAMA Network Open.*

eTable 2. Performance of the Final Prediction Model Across Prediction Periods

	SNUH internal validation dataset			BMC external validation dataset		
	Prediction of massive transfusion 10 minutes in advance	Prediction of massive transfusion 15 minutes in advance	Prediction of massive transfusion 20 minutes in advance	Prediction of massive transfusion 10 minutes in advance	Prediction of massive transfusion 15 minutes in advance	Prediction of massive transfusion 20 minutes in advance
AUROC	0.972	0.969	0.962	0.943	0.957	0.912
AUPRC	0.571	0.555	0.492	0.370	0.458	0.393
sensitivity	0.905	0.913	0.909	0.818	0.909	0.900
specificity	0.905	0.911	0.904	0.849	0.940	0.877
PPV	0.159	0.169	0.154	0.107	0.256	0.134
NPV	0.997	0.998	0.997	0.995	0.997	0.997

Abbreviations: AUPRC, area under the precision-recall curve; AUROC, area under the ROC curve; BMC, Boramae Medical Center; NPV, negative predictive value; SNUH, Seoul National University Hospital; PPV, positive predictive value.

© 2022 Lee SM et al. *JAMA Network Open.*

eFigure 1. Overview of Data Engineering and Integration

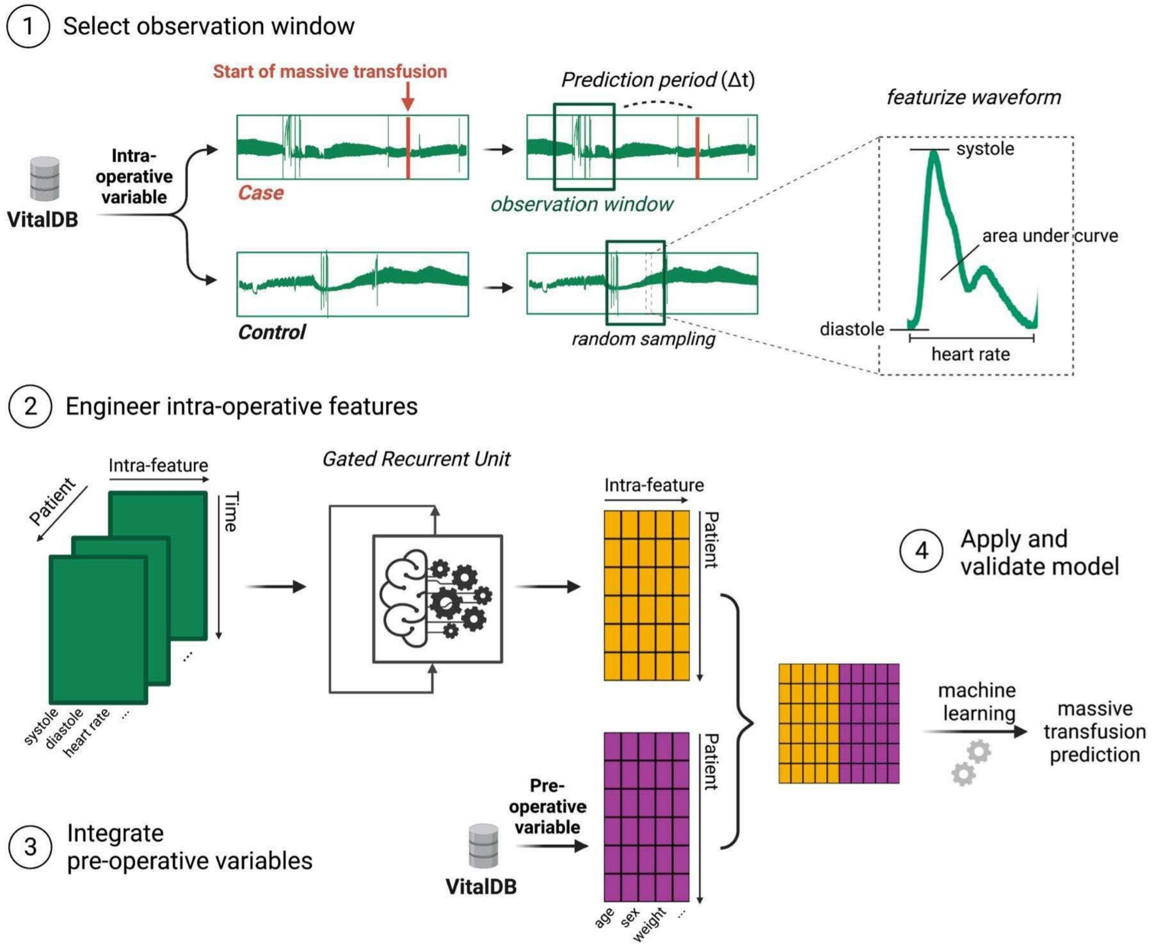

Intraoperative features were downloaded from vitalDB and segmented depending on observation window and prediction period. Feature factorization is performed to extract area under curve, systole, diastole, and heart rate. To integrate time series intraoperative vital sign with preoperative features GRU transforms time series vital sign into regular feature matrix. Then intraoperative and preoperative features are concatenated to run massive transfusion prediction model.

eFigure 2. The Performance of Preoperative and Real-Time Intraoperative Prediction Model Evaluation in BMC External Validation Dataset

a) Area under the receiver characteristic curve (AUROC)

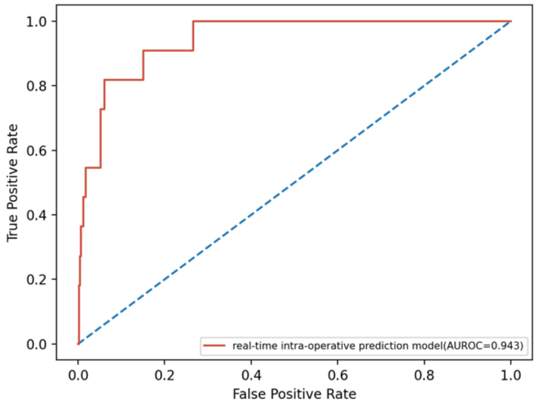

b) Area under the precision-recall curve (AUPRC)

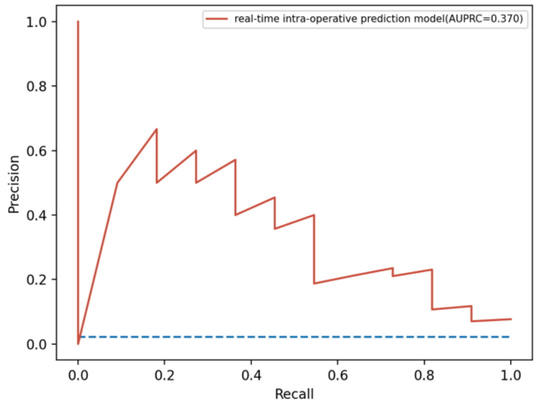

Abbreviations: BMC, Boramae Medical Center.

© 2022 Lee SM et al. *JAMA Network Open.*

eFigure 3. Massive Transfusion Index Analysis in SNUH Study Population

a) The occurrence of massive transfusion according to the deciles of the massive transfusion index

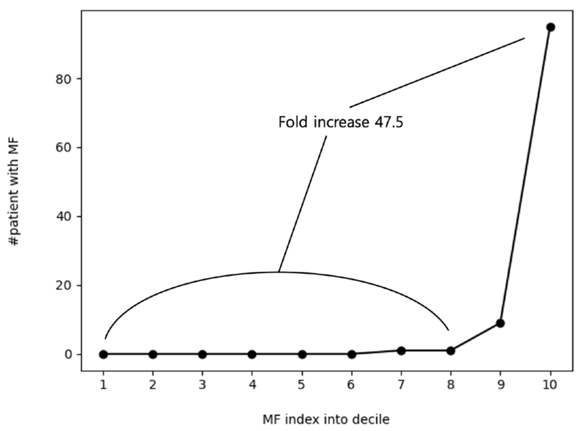

b) The distributional difference in the massive transfusion index between cases and controls

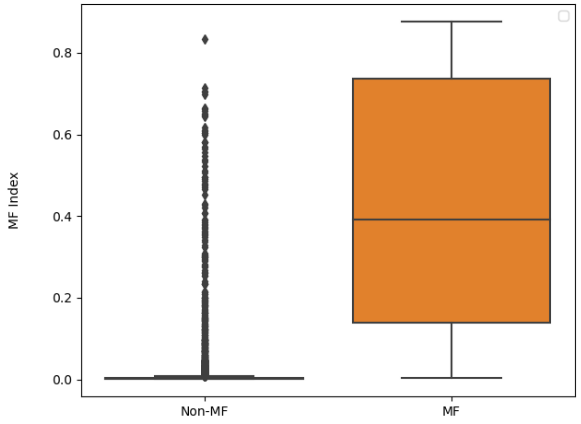

Abbreviations: SNUH, Seoul National University Hospital

© 2022 Lee SM et al. *JAMA Network Open.*

eFigure 4. Massive Transfusion Index Analysis in BMC Study Population

a) The occurrence of massive transfusion according to the deciles of massive transfusion index

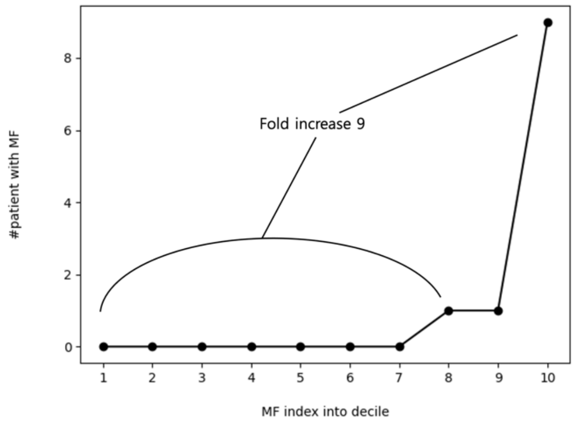

b) The distributional difference of massive transfusion index between case and control

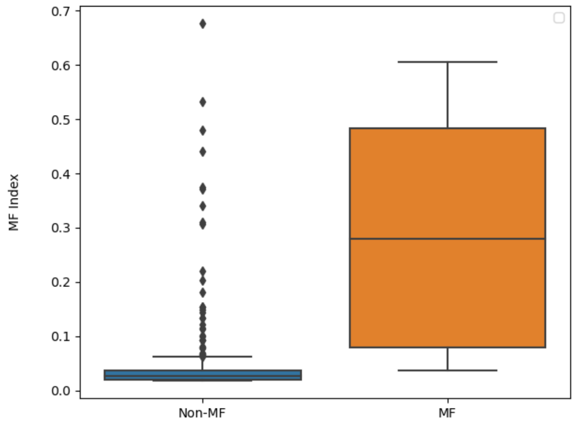

Abbreviations: BMC, Boramae Medical Center

© 2022 Lee SM et al. *JAMA Network Open.*
